# ADS-B Crowd-Sensor Network and Two-Step Kalman Filter for GNSS and ADS-B Cyber-Attack Detection

**DOI:** 10.3390/s21154992

**Published:** 2021-07-22

**Authors:** Mauro Leonardi, Gheorghe Sirbu

**Affiliations:** Department of Electronic Engineering, Tor Vergata University of Rome, 00133 Rome, Italy; gheorghe.sirbu@uniroma2.it

**Keywords:** ADS-B, GNSS, security, intrusion detection, spoofing, jamming, crowd sourced network, localization, EKF

## Abstract

Automatic Dependent Surveillance-Broadcast is an Air Traffic Control system in which aircraft transmit their own information (identity, position, velocity, etc.) to ground sensors for surveillance purposes. This system has many advantages compared to the classical surveillance radars: easy and low-cost implementation, high accuracy of data, and low renewal time, but also limitations: dependency on the Global Navigation Satellite System, a simple unencrypted and unauthenticated protocol. For these reasons, the system is exposed to attacks like jamming/spoofing of the on-board GNSS receiver or false ADS-B messages’ injection. After a mathematical model derivation of different types of attacks, we propose the use of a crowd sensor network capable of estimating the Time Difference Of Arrival of the ADS-B messages together with a two-step Kalman filter to detect these attacks (on-board GNSS/ADS-B tampering, false ADS-B message injection, GNSS Spoofing/Jamming). Tests with real data and simulations showed that the algorithm can detect all these attacks with a very high probability of detection and low probability of false alarm.

## 1. Introduction

The Automatic Dependent Surveillance Broadcast (ADS-B) system is considered to be the backbone of the future air traffic control system [1,2]. Today, more than 80% of commercial aircraft are currently equipped with ADS-B hardware [3]. It is a dependent and cooperative surveillance system in which the aircraft has an ADS-B transponder mounted on-board and continually broadcasts its state vector obtained from the Global Navigation Satellite System (GNSS) positioning equipment to anyone equipped with an ADS-B receiver [4]. The transponder uses the radar Mode S protocol to broadcast this information through the digital data link on the shared L-band channel. The Mode S protocol foresees 120 μs messages that contain a data block which is 112 bits long (112 μs), and an additional 8 μs preamble for synchronization. The messages use the Pulse Position Modulation (PPM), with a pulse length of 0.5 μs and the channel has a maximum speed of 1 Mbit/s. In nominal conditions, the ADS-B messages are received by the ground-based receivers and used to generate traffic images on the controller’s display.

**Figure 1:**
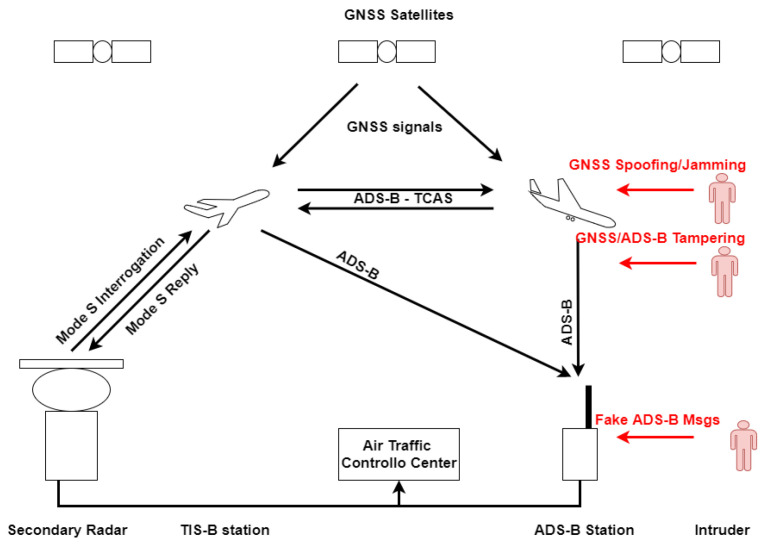
ADS-B system architecture. Possible attacks are in red.

Figure 1 shows the ADS-B system architecture: the aircraft computes its position exploiting the GNSS and broadcasts messages containing the state information which are received by other aircraft, and by the ADS-B ground station for ATC operations. Possible attackers are highlighted in red; they could jam or spoof the GNSS signals, tamper the on-board GNSS receiver or the ADS-B transponder, or, finally, directly inject false ADS-B messages in the system.

The ADS-B system has many advantages compared to the classical surveillance radars; for example, the ground stations can be installed in almost any location, resolving the problem of black spots in remote areas where a radar station cannot be installed; it has an easy and low-cost implementation, and increased accuracy of location data allows for smaller minimum aircraft separations, and therefore higher capacity. It has also some important limitations such as: the dependency on the GNSS (which suffers from Jamming and Spoofing that could corrupt, damage or interfere with the positioning information), and the very simple protocol that is not encrypted and without any authentication.

Due to these limitations, different cyber-attacks can be used against the ADS-B system, and they can be classified as follows [5]:

- ADS-B Eavesdropping, i.e., listening to the transmission of the broadcasted messages since there is a lack of encryption: it is impossible to prevent without applying encryption and, of course, it is impossible to be detected;
- ADS-B channel Jamming, i.e., transmitting intentionally signals with a jammer in the RF channel in order to deny the communication: this type of attack may create denial-of-service (DoS) problems;
- ADS-B message injection (or spoofing), i.e., transmitting malicious signals, with a power slightly higher than a legitimate ADS-B signal, which contain misleading information, for example transmitting a sequence of messages representing a fake aircraft on a realistic trajectory;
- ADS-B Message deletion by SSR reply Garbling: deletion of some or all the information contained in a message by exploiting constructive or destructive signal interference;
- ADS-B Message modification, i.e., modifying messages of legitimate nodes by overshadowing, bit flipping or with a combination of deletion and injection;
- GNSS Spoofing/Jamming, i.e., transmitting high power noise or signal on the GNSS channel producing false or wrong position information or reducing the GNSS availability;
- Aircraft ADS-B transponder or GNSS receiver tampering that is not authorized access to the on-board device that, in principle, allows different types of attacks—for example, the injection of false position in the navigation system or the manipulation and transmission of ADS-B message containing false information.

To mitigate some of these ADS-B related risks, various techniques were proposed in the last few years. As described, ADS-B messages are unencrypted and unauthenticated, and this leads to the possibility for an attacker to modify messages or inject fake messages. Encryption and authentication on the ADS-B protocol were proposed to overcome eavesdropping and message injection, like in [5,6,7,8], but they need a protocol modification. Authentication by RF and Low Level fingerprinting, without protocol modification, are proposed in [9,10,11,12,13]. In [14], the authors also propose a method to improve the ADS-B system security by introducing a physical layer protocol evolution for the introduction of an authentication scheme fully compliant with the current standard and exploiting the phase modulation of the signals. In [15], the tracking of the different sensor clocks is proposed, by the use of Time Difference of Arrival (TDOA), of ADS-B messages to check the veracity of the position information contained in the ADS-B messages and to prevent false ADS-B data injection. Many other works in the past proposed the use of TDOA and Multilateration (MLAT) to verify the veracity of the ADS-B data starting from [16]. ADS-B jamming and message deletion vulnerabilities cannot be reduced with encryption or data verification and are closely related to the ADS-B receiver hardware; they require anti-jamming techniques—see, for example, [11,17,18], or [19,20] where the authors investigate network-wide countermeasures based on redundant coverage. Concerning the GNSS spoofing, there are different types of attacks, as discussed in [21], that threaten the actors that exploit the GNSS. Methods against spoofing attacks include authentication of the GPS signal [22,23], spatial processing aiming to nullify the signal coming from the direction of the incoming attack [24,25], and other methods that include machine learning based techniques for the detection of the spoofing attack [26,27]. These methods are applied on the GNSS receiver and are out of the scope of this work. Refs. [16,28,29] report some methods that exploit multilateration (MLAT), for the integrity check of GNSS derived information that can be used in ADS-B sensors’ networks. MLAT provides an independent position information through hyperbolic localization, allowing for the detection of GNSS spoofing attacks.

More recent works also demonstrate that is possible to apply the MLAT algorithm for detecting malicious ADS-B transmitters [30,31]; they also propose to improve the MLAT performance by optimizing the network geometry (using genetic algorithms) and smoothing the measurements by the use of Kalman filters. Finally, in [32], it was shown that it is possible to achieve precise TDOA estimation also using low-cost ADS-B receivers in the MLAT sensor network.

The main disadvantages of MLAT are that it requires a minimum of four ground stations to calculate the position to be verified, and the system must invert a strong-not-linear and ill-posed system that produces numerical instability in the solution [33,34].

Finally, tampering the on-board GNSS receiver or the ADS-B transponder to inject false information has the same effect of GNSS spoofing and can be mitigated in the same way as before, using position consistency check.

Fortunately, nowadays, there are different types of ADS-B sensors and sensor networks that can be utilized for ADS-B message information checking. For example, there are open crowd-sourced networks, like *FlightRadar24* [35] or *OpenSky Network* [36] that are becoming more and more used in the ATC community. In the following, we propose the use of these networks to detect the most important threats on the ADS-B and GNSS channels, in particular:

- we analyze the effects on the ADS-B message information and on the ADS-B sensor network of the different types of threats, developing mathematical models to represent the following attack types: ADS-B message injection, GNSS jamming, GNSS spoofing, and on-board device tampering;
- we propose a method that, exploiting the network measurements, is able to detect these types of attack, without solving the MLAT problem and also taking into account the information about the expected kinematic state of each aircraft.

The proposed method exploits synchronized ADS-B sensors capable of measuring the TDOA of the ADS-B messages, and it was evaluated with real data coming from the OpenSky Network. For any ADS-B message, two different sources of information related to the aircraft position are available in the network: the TDOA measurements and the GNSS estimated position encoded on the ADS-B message. Exploiting a Kalman Filter [37,38], the aircraft kinematic model can be derived and any type of measurements can be integrated to have a better estimation of the aircraft position. Moreover, the Kalman filter has the ability to forecast the expected measurements and, comparing these with the incoming ones, it is possible to check the consistency between the aircraft estimated state and the measurements coming from the GNSS and the station network.

With this approach, it is not necessary to solve the MLAT problem, as done in the previously mentioned works, because the test was done directly in the measurement domain. This allows for performing the test also in case of bad geometry or with less than four stations in view from the aircraft.

In particular, here, we propose a two-step Extended Kalman Filter (EKF), with two consecutive updating phases and two different consistency checks (on the track kinematic and on the emitter position).

Simulations and trials show that the combination of the two tests is able to detect a small deviation from the aircraft predicted trajectory and also to detect false tracks injected by GNSS or ADS-B spoofers that have realistic but fake trajectory.

Last, but not least, considering that, in the last few years, GNSS attacks (in particular the spoofing attack) are increasing, the proposed method that uses the fighting aircraft together with the ground based ADS-B network as a large and pervasive opportunity sensor network to monitor and detect GNSS attacks can improve the general GNSS security also in other fields of application.

In the following sections, the different types of threats are described and modeled, the proposed detection method is derived and evaluated with simulations, using real data coming from the OpenSky Network, and, finally, some conclusions are reported.

## 2. Threat Models

As mentioned before, different types of attacks can affect the elements of the ADS-B system. In this work, we will focus on the following four threats:

- GNSS jamming;
- GNSS spoofing;
- ADS-B spoofing with fake messages injection;
- On-board ADS-B transponder or GNSS receiver tampering.

In this section, we describe the main threats and discuss the effects on the ADS-B system and on the sensor network; finally, we develop a mathematical model to represent the effect of each threat on the ADS-B system.

### 2.1. GNSS Jamming Attack

A jamming attack is used against the GNSS receiver to reduce ADS-B aircraft localization performance or to totally deny the localization service. The jammer transmits high power signals in the band of the GNSS to reduce as much as possible the sensitivity of the receiver, improving the noise floor and reducing the performance of the receiver. Moreover, if the jammer is very close to the receiver under attack, the signal-to-noise ratio becomes so small (or the receivers amplifiers go in their saturation zone) that the receiver is not able to decode the GNSS signals, with a total denial of the service.

In this paper, we assume that this second condition doesn’t happen: considering flying objects, the receiver is assumed far enough from the jammer to avoid the saturation zone of its components. Consequently, we propose here a model to represent only the position accuracy degradation of the receiver when it is not close to its saturation. The derivation of a more complex model, taking into account nonlinear effects that are dependent on the receiver hardware and on the base-band processing of the different types of GNSS receivers, is out of the scope of this work.

The GNSS jamming effect on the ADS-B system can be modeled with an improvement of the error on the position encoded in the ADS-B messages. Calling $x={\left(x,y,z\right)}^{T}$ the vector containing the true aircraft position, the ADS-B aircraft position is:

(1) $$x_{ADS-B}=x+n_{ADS-B}$$

where $n_{ADS-B}$ is the three elements ADS-B error vector representing the 3D error on the ADS-B position due to the GNSS localization. In a nominal condition, for the sake of simplicity, this error can be assumed with zero mean and covariance matrix given by:

(2) $$Q_{ADS-B}=\left[\begin{array}{ccc} \sigma_{x}^{2} & 0 & 0\\ 0 & \sigma_{y}^{2} & 0\\ 0 & 0 & \sigma_{z}^{2} \end{array}\right]=\sigma_{ADS-B}^{2}\left[\begin{array}{ccc} 1 & 0 & 0\\ 0 & 1 & 0\\ 0 & 0 & 1 \end{array}\right]$$

where we have assumed that the noise components are uncorrelated with each other, and, in the second equality, that the error has the same distribution for the three components.

By fixing the $\sigma_{ADS-B}$ value, it is possible to over-bound the position error for a GNSS receiver on-board. Moreover, the position errors in GNSS systems depend on two factors [39]: the geometry of the GNSS satellite during the measurements (usually referred as Dilution Of Precision—DOP) and the User Equivalent Range Error (UERE) that takes into account the measurement error of the receiver:

(3) $$\sigma_{x,y,z}=f\left(UERE\right)$$

Under common conditions, the function *f* that takes into account the system geometry is considered linear, and this relationship can be simplified in $\sigma_{x,y,z}=DOP_{x,y,z}\cdot UERE$.

The UERE is the sum of the GNSS pseudorange measurements’ error contributions—for example, the sum of errors due to the ionosphere, the troposphere, and the measurement process in the receiver:

(4) $$UERE=\sigma_{iono}+\sigma_{tropo}+\ldots +\sigma_{rx}$$

Usually, the GNSS receiver noise ($\sigma_{rx}$) is smaller than the other contributions, but, in case of jamming, it increases and can become higher than the others. Let us suppose to have a jammer with a given transmitter power ($P_{J}$) and a given antenna gain ($G_{J}$). Using the Friis equation [40,41], it is possible to compute the corresponding received power ($P_{j}^{r}$):

(5) $$P_{j}^{r}=G_{J}G_{r}\left(\theta_{J},\phi_{J}\right){\left(\frac{\lambda }{4\pi R_{J}}\right)}^{2}P_{J}$$

where $R_{J}$ is the jammer–aircraft range, and $G_{r}\left(\theta_{J},\phi_{J}\right)$ is the receiver antenna gain in the direction of the jammer. If $P_{j}^{r}$ is higher than the receiver amplifier saturation threshold, $P_{sat}$, the reception of the GNSS signal is inhibited, and no navigation solution is carried out from the receiver. As mentioned before, if the jammer received power is also lower than the saturation level, the receiver performances are reduced. Fixing the received power for the signals coming from the satellites, $P_{T}^{r}$, the signal-to-interference-plus-noise ratio (SINR) becomes:

(6) $$SINR=\frac{P_{T}^{r}}{N+P_{J}^{r}}=\frac{P_{T}^{r}}{N+G_{J}G_{r}\left(\theta_{J},\phi_{J}\right){\left(\frac{\lambda }{4\pi R_{J}}\right)}^{2}P_{J}}≃kR_{J}^{2}.$$

The last equality is valid in case $N\ll P_{J}^{r}$; under this condition, the relationship between the jammer distance and the SINR is quadratic (apart from a constant factor *k*). Recalling that $\sigma_{rx}$ depends on the SINR with the following relationship [42]:

(7) $$\sigma_{rx}\left(SINR\right)≅\frac{1}{\beta \sqrt{2SINR}}.$$

During a jamming attack, this term becomes bigger than the other contributions in the UERE budget Equation (4) and using Equations (3) and (7), it is possible to infer a value to over-bound the GNSS position error in case of a jammer at a given distance $R_{J}$:

(8) $$\sigma_{ADS-B}\left(SINR\right)=f\left(\cdot \left(const+\sigma \left(SINR\right)\right)\right)=a+\frac{1}{\beta_{2}\sqrt{2kR_{J}^{2}}}=a+b/R_{J}$$

where *a* is a constant that takes into account all the other error effects, and *b* gives the linear relationship between the distance of the jammer and the GNSS error magnitude (it depends on the jammer parameters, such as the transmitted power, type of transmitted signal, etc.). In the last equality, as stated before, it is assumed that the *f* function is a linear function as is usually assumed for the computation of the Dilution of Precision in the GNSS system [39]. It follows that, for a GNSS jamming attack, we can assume that the ADS-B message will contain position data with errors on the three components ($n_{ADS-B}$) that are still with zero mean but with higher standard deviations that decrease linearly with the jammer distance, Equation (2) becomes:

(9) $$Q_{ADS-B}^{Jamming}=(\sigma_{ADS-B}^{2}+\overset{\sigma_{Jamming}^{2}}{\overset{︷}{{\left(a+b/R_{J}\right)}^{2}}})\cdot \left[\begin{array}{ccc} 1 & 0 & 0\\ 0 & 1 & 0\\ 0 & 0 & 1 \end{array}\right]$$

### 2.2. GNSS Spoofing Attack

GNSS spoofing is the transmission of fake GNSS signals containing false information. The effect on the receiver is more dangerous compared with the jamming for at least two reasons: it is more difficult to detect by the user, and the receiver uses the fake signals carrying out wrong positions that can have huge biases and can also follow a fake trajectory [43,44].

On the ADS-B system, GNSS spoofing means the ability for an attacker to introduce fake positions in the aircraft under attack. This effect can be modeled adding a bias on the aircraft position, maintaining the same standard deviation for positional error:

(10) $$x_{ADS-B}=x_{fake}+n_{ADS-B}=x+b_{GNSS}+n_{ADS-B}$$

where $b_{GNSS}$ represents the bias due to the spoofing, i.e., the vector representing the different between the real and the fake position.

### 2.3. ADS-B Spoofing

ADS-B spoofing includes different possible attacks; in this work, we refer to the most dangerous case: the injection in the Mode S channel of legacy formatted ADS-B messages containing false information. These messages are received from the ADS-B station and are not distinguishable from the correct ones. This can produce two main effects on the ADS-B system:

- the appearing of “ghost” aircraft, in case of fake messages containing data of airplanes not yet present in the coverage area of the ADS-B station;
- the appearing of wrong ADS-B position data incoherent with the other position coming from the real messages, if the aircraft is present in the coverage of the ADS-B system.

In any case, the injection of fake ADS-B messages with wrong positional data can be seen as the introduction of a bias on the ADS-B position, as already done in the case of GNSS spoofing:

(11) $$x_{ADS-B}=x_{fake}=x+b_{ADS-B}$$

The difference with GNSS spoofing is that, unlike GNSS spoofing that affects only the aircraft close to the spoofer, ADS-B message injection affects only the station under attack and can introduce any number of fake airplanes in any possible position.

### 2.4. On-Board Tampering Attack

On-Board tampering consists of the replacement, or the manumission, of the on-board hardware or software to inject false information in the on-board devices; for example, replacing the GNSS unit with an emulator that carries out false position or hacking of the ADS-B transponder to transmit fake ADS-B messages. In any case, this threat can be modeled as the previous threats, introducing a bias in the position transmitted by the ADS-B transponder:

(12) $$x_{ADS-B}=x_{fake}=x+b_{tamp}$$

### 2.5. Discussion on the Threats Models

The introduced models are used in the following section to emulate the possible attacks on the ADS-B system. The first model (GNSS spoofing) is the only one that introduces additive noise on the ADS-B position. This noise produces more noisy tracks for the aircraft close to the jammer. This effect can be seen in the same moment on one or more aircraft and for the whole duration of the attack. The mathematical model for the other three threats is the same, but the effects on the whole ADS-B system are different:

- in case of GNSS spoofing, the bias appears only for the aircraft close to, or in the coverage of, the spoofer, typically changing the position of the aircraft with a fake one. Moreover, the fake position is the same for all the attacked aircraft. Observing the whole traffic, under this attack, aircraft close to each other and near the spoofer instantly change their positions to a new fake one, equal for all the airplanes. Note that GNSS Spoofing can be very dangerous because the on-board navigation systems also use the GNSS; it means that both the pilot and the controller are deceived, this may cause the total loss of the control of the aircraft;
- in case of ADS-B spoofing, one or more ghost aircraft, having false positions, can appear in any place in the world, and not necessarily near the ADS-B stations that receive the signals; in general, there is no correlation between the ADS-B spoofer and the ghost airplanes’ position. Moreover, the fake aircraft can have realistic trajectory. If the injected airplanes are already present and tracked by the ADS-B sensor network, this attack produces outliers or big steps in the airplane tracks. If the injected airplanes are not yet tracked, totally new (and fake) tracks appear in the ADS-B system;
- in case of on-board device tampering, only the aircraft under attack transmits fake positions. This attack can produce steps on the airplane track or, if well-designed, can slightly change the airplane positions to produce a false track, without unexpected steps (for example, the aircraft can change its real trajectory continuing transmitting the expected one to hide a hijacking).

Finally, concerning the GNSS spoofing and jamming, it is important to note that they are used for many reasons in different fields, usually to inhibit its uses; this means that the aircraft can be a secondary victim of an attack designed for other scopes. On the other hand, the aircraft and the ADS-B system can be seen as a detector for any GNSS spoofing and jamming attack also if it is designed for other scopes.

## 3. Attack Detection Algorithm

As mentioned before, we propose to monitor the ADS-B data exploiting a crowd-sensor network able to perform TDOA estimation of the received ADS-B messages: the idea is to combine the network measurements with the incoming ADS-B data to perform some conformance tests.

Recently, the OpenSky Network released some data-sets containing the TOA of the ADS-B messages. Moreover, many of these stations are GPS synchronized, allowing the TDOA localization of the aircraft [45]. Here, we propose to use TDOA measurements of ADS-B messages not to have an independent estimation of the aircraft position but to verify the consistency of the two sources of information without solving the MLAT problem. This allows for checking the ADS-B data also when less than four stations are present and without solving the MLAT problem that many times can be ill-conditioned.

Moreover, we propose to also test the consistency of the incoming measurements with the historical information and the expected kinematic model for the aircraft. In fact, exploiting Kalman filters, it is possible to use the aircraft expected kinematic model, the statistical knowledge of the measurements and the past measurements to predict the future target position and to predict the expected measurements to be compared with the incoming ones to verify their consistency.

In the following sections, we will derive, firstly, the model and the statistics to test the TDOAs and ADS-B position consistency, and then we extend these models to be used into an EKF.

### 3.1. Position Check by the Use of TDOAs

Assuming to have *M* synchronized stations able to measure the TOA of the messages, for each received message, it is possible to write the following equation:

(13) $$TOA_{i}^{k}=\frac{∥x_{i}-x^{k}∥}{c}+t_{0}^{k}+n_{i}$$

where $x_{i}$ represents the position of the station *i*, and $x^{k}$ represents the position of the aircraft *k*, *c* is the speed of light, $t_{0}^{K}$ is the time of transmission of the message, and $n_{i}$ represents the TOA measurement errors of the station *i*. Forming the differences between two stations, it is possible to cancel the unknown $t_{0}^{k}$:

(14) $$TDOA_{i,j}^{k}=\frac{∥x_{i}-x^{k}∥-∥x_{j}-x^{k}∥}{c}+n_{i}-n_{j}$$

Now, fixing a reference station, (M−1) independent TDOAs can be computed and, using the matrix notation, we have:

(15) $${\mathrm{TDOA}}^{k}=f\left(x_{1},\ldots ,x_{M},x^{k}\right)+n_{TDOA}$$

where ${\mathrm{TDOA}}^{k}$ is the vector containing the (M−1) measurement, $n_{TDOA}$ is the M−1 measurements error vector, having zero mean and covariance matrix:

(16) $$Q_{TDOA}=\left[\begin{array}{ccccc} \sigma_{1}^{2}+\sigma_{M}^{2} & \sigma_{M}^{2} & \ldots & \sigma_{M}^{2}\\ \; & \; & \ldots & \; & \\ \sigma_{M}^{2} & \ldots & \sigma_{i}^{2}+\sigma_{M}^{2} & \sigma_{M}^{2}\\ \; & \; & \ldots & \; & \\ \sigma_{M}^{2} & \ldots & \sigma_{M}^{2} & \sigma_{M-1}^{2}+\sigma_{M}^{2} \end{array}\right]$$

where $\sigma_{1}^{2},\ldots ,\sigma_{M}^{2}$ are the TOA measurement variances for each station.

For the same aircraft, at the same instant, the GNSS derived aircraft position is encoded in the ADS-B message and available in the ADS-B ground station: it can be denoted with $x_{ADS-B}^{k}$, as defined in Equation (1). The ADS-B position corresponding to the expected TDOA measurement vector can be computed exploiting Equation (14) and Equation (15):

(17) $${\mathrm{TDOA}}_{ADS-B}^{k}=f\left(x_{1},\ldots ,x_{M},x_{ADS-B}^{k}\right)=f\left(x_{1},\ldots ,x_{M},x^{k}+n_{ADS-B}\right)$$

and linearizing near the real target position, $x^{k}$, it is possible to obtain:

(18) $${\mathrm{TDOA}}_{ADS-B}^{k}=f\left(x_{1},\ldots ,x_{M},x^{k}\right)+\frac{\partial f(x_{1},\ldots ,x_{M},x^{k})}{\partial x}{|}_{\left(x=x^{k}\right)}\cdot n_{ADS-B}^{k}$$

Finally, forming the difference between Equation (18) and Equation (15), we obtain a vector representing the difference between the ADS-B expected TDOA and the measured TDOA:

(19) $$\epsilon ={\mathrm{TDOA}}_{ADS-B}^{k}-{\mathrm{TDOA}}^{k}=\frac{\partial f(x_{1},\ldots ,x_{M},x)}{\partial x}{|}_{\left(x=x^{k}\right)}\cdot n_{ADS-B}^{k}-n_{TDOA}$$

Calling F the Jacobian of f, we have:

(20) $$\epsilon =F\cdot n_{ADS-B}^{k}-n_{TDOA}$$

It follows that, in an ideal case, without measurement noises and ADS-B position errors, ϵ is an (M−1) vector composed of zeros. In the presence of noises and position errors, ϵ is a random vector that depends only on the measurement noises, the ADS-B errors, and on the matrix F.

Assuming Gaussian distributed noises and errors, the entries of ϵ will be linear combinations of Gaussian variables, still Gaussian distributed with covariance matrix given from the following equation:

(21) $$Q=FQ_{ADS-B}F^{T}+Q_{TDOA}$$

Finally, the normalized norm of ϵ, $w=\epsilon^{t}Q^{-1}\epsilon$ can be used to test the consistency of the ADS-B position and TDOA measurements. In nominal conditions (that is absence of any anomalies), called $H_{0}$ hypothesis, recalling that the normalized norm is the sum of (M−1) Gaussian random variables, *w* is chi-square distributed [46]:

(22) $$H_{0}:w∼\chi^{2}\left(M-1,0\right)$$

In the case of ADS-B spoofing/on-board tampering/GNSS spoofing ($H_{1}$ hypothesis), assuming the presence of a bias on the ADS-B position in the messages, Equation (20) becomes:

(23) $$\epsilon =F\cdot \left(n_{ADS-B}^{k}+b_{ADS-B}^{ADS-B/GNSS/tamp}\right)-n_{TDOA}^{k}$$

In this case, the distribution for *w* will be a not central chi-square distribution with (M−1) degrees of freedom with parameters [46]:

(24) $$H_{1}:w∼\chi^{2}(M-4,∥F\cdot b_{ADS-B/GNSS/tamp}{∥}_{2}^{2})$$

Unfortunately, the vector $b_{ADS-B/GNSS/tamp}$ is projected into the TDOA-space through the matrix F, and, if F has a null-space that is not null, some biases could be undetectable. In any case, the resulting norm can be reduced by this projection and there is not an easy way to find a lower bound for the non-central parameter of the chi-square distribution. It follows that it is impossible to find a general way to fix the probability of missed detection: it depends on the combination of ADS-B bias (magnitude and direction) and geometrical distribution of the sensor stations used for the test. Finally, in case of GNSS Jamming, the distribution of the parameters will not be a chi-square distribution and cannot be computed explicitly. For these reasons, the algorithm performance in terms of Missed Detection (or Detection Probability) should be evaluated via Monte Carlo simulations for each specific geometrical configuration of the networks.

In any case, the distribution of *w* in nominal condition ($H_{0}$ hypothesis) is always known (knowing the ADS-B position error and the TDOA measurement noise parameters) and can be used to at least fix the Probability of False Alarm of the test, finding a threshold for the *w* parameter:

(25) $$P_{fa}=\int_{t_{P_{fa}}}^{\infty }\chi^{2}\left(M-1,\hat{\lambda }\right)$$

If that threshold is overcome, the presence of an anomaly can be declared.

### 3.2. Two Step Detection by the Use of Kalman Filtering

The approach described in the previous section only checks the ADS-B position information with the TDOA measurements, without taking into account the possible kinematic model for the target. In this section, we extend the concept to be implemented in a tracking algorithm, able to also use the kinematic parameters and the past measurements to forecast the expected TDOA for any new incoming message of the airplane.

Assuming an aircraft with a constant velocity in the short time, and exploiting all the statistical knowledge of the observations (ADS-B position error and TDOA measurement noise), it is possible to set a Discrete Time EKF. The aircraft state transition model is:

(26) $$s^{k}\left(t_{n+1}\right)=Gs^{k}\left(t_{n}\right)+w\left(t_{n}\right)$$

where $s^{k}\left(t_{n}\right)$ is the state vector of the aircraft *k* at time instant $t_{n}$, defined as:

(27) $${\left(s^{k}\right)}^{T}=\left[\begin{array}{c} x,y,z,v_{x},v_{y},v_{z} \end{array}\right]$$

where $x,y,x,v_{x},v_{y},v_{z}$ represents the position and velocity components of the aircraft; the transition matrix, G, is defined as:

(28) $$G=\left[\begin{array}{cccccc} 1 & 0 & 0 & T & 0 & 0\\ 0 & 1 & 0 & 0 & T & 0\\ 0 & 0 & 1 & 0 & 0 & T\\ 0 & 0 & 0 & 1 & 0 & 0\\ 0 & 0 & 0 & 0 & 1 & 0\\ 0 & 0 & 0 & 0 & 0 & 1 \end{array}\right]$$

and $w(t_{n})$ represents the process noise. Considering the measurement models, two measurements must be considered: the use of position encoded on the ADS-B message $x_{ADS-B}^{k}\left(t_{n}\right)$, and the TDOAs measurements, ${\mathrm{TDOA}}^{k}$ (recalling Equation (1) and Equation (15)):

(29) $$x_{ADS-B}^{k}\left(t_{n}\right)=\left[\begin{array}{cccccc} 1 & 0 & 0 & 0 & 0 & 0\\ 0 & 1 & 0 & 0 & 0 & 0\\ 0 & 0 & 1 & 0 & 0 & 0 \end{array}\right]s^{k}\left(t_{n}\right)+n_{ADS-B}\left(t_{n}\right)=F_{ADS-B}s^{k}\left(t_{n}\right)+n_{ADS-B}\left(t_{n}\right)$$

$${\mathrm{TDOA}}^{k}\left(t_{n}\right)=f\left(s\left(t_{n}\right)\right)+n_{TDOA}\left(t_{n}\right)$$

Remembering Equation (14) and Equation (15), the Jacobian of the function f can be easily computed for the EKF application:

(30) $$F_{TDOA}=\frac{\partial f(s\left(t_{n}\right))}{\partial s(t_{n})}=\left[\begin{array}{cc} F & 0_{3x3} \end{array}\right]$$

where F is introduced in the previous section. Now, the EKF can be applied to estimate the aircraft position exploiting both the ADS-B encoded position and TDOA measurements; here, we propose to use a two step updating process: the first update is done with the ADS-B data and the second update is done with the TDOA measurements. In this way, two innovations (the difference between the expected measurements and the incoming measurements) are computed (w.r.t. the ADS-B data and w.r.t. TDOAs) and can be used to produce two different conformance checks: one on the conformance of the new ADS-B data with the track information, and the other on the conformance of the track with independent TDOA measurements. The steps of the proposed algorithm are reported in Table 1.

It is important to note that this approach allows, at the same time, to test the data and measurement coming from the ADS-B messages and from the sensor measurement, and to have a more accurate estimation of the target position. In the algorithm, three different thresholds were introduced: $th_{1}$ is used to check the ADS-B data/track consistency, using the normalized norm of the first step innovation, $th_{2}$ is used to check the consistency of the TDOA/track consistency using the normalized norm of the second step innovation and $th_{3}$ is used to decide whether to update the track states with the TDOA measurements or not. The normalized norms are computed using the innovation vectors and their covariance matrices, in the same way as illustrated in the previous section.

The test parameters become:

(31) $$\begin{array}{c} w_{ADS-B}^{k}\left(t_{n}\right)={\left(y_{ADS-B}^{k}\left(t_{n}\right)\right)}^{t}R_{ADS-B}\left(t_{n}\right)y_{ADS-B}^{k}\left(t_{n}\right)\\ w_{TDOA}={\left(y_{TDOA}^{k}\left(t_{n}\right)\right)}^{t}R_{TDOA}\left(t_{n}\right)y_{TDOA}^{k}\left(t_{n}\right) \end{array}$$

where $y_{ADS-B}^{k}\left(t_{n}\right)$ is the ADS-B position innovation for the aircraft *k* at time $t_{n}$, and $y_{TDOA}^{k}\left(t_{n}\right)$ is the TDOA innovation for the same aircraft and time instant, having covariance matrix (computed by the EKF) $R_{ADS-B}\left(t_{n}\right)$ and $R_{TDOA}$, respectively (see Table 1 for their definitions).

Distributions for the $H_{0}$ hypothesis, to fix the Probability of False Alarm, can be still computed as described in the previous section, and becomes:

(32) $$\begin{array}{cc} H_{0}:w_{ADS_{B}} & ∼\chi^{2}\left(3,0\right)\\ w_{TDOA} & ∼\chi^{2}\left(M-1,0\right) \end{array}$$

It is easy to understand that $w_{ADS-B}$ is suitable to detect any attack that introduces a step in the trajectory (e.g., GNSS spoofing when switched on, GNSS Jamming, ADS-B spoofing of aircraft already present in the coverage area, simple ADS-B spoofing producing a step in the trajectory, simple tampering producing a step in the trajectory, etc.). Smarter attacks, e.g., ADS-B spoofing with realistic false track injection, or tampering with the introduction of a fake trajectory with a smooth transition from the real one, cannot be detected. In this second case, the $w_{TDOA}$ test is still able to detect the aircraft deviation from the declared trajectory (on board tampering or false aircraft injection; in general smooth but growing deviation of ADS-B positions from the real one) using independent TDOA measurements.

Moreover, as mentioned before, observing all of the traffic, it is possible to distinguish between on-board tampering, GNSS attacks, and ADS-B attacks.

## 4. Evaluation with Real Data

**Figure 2:**
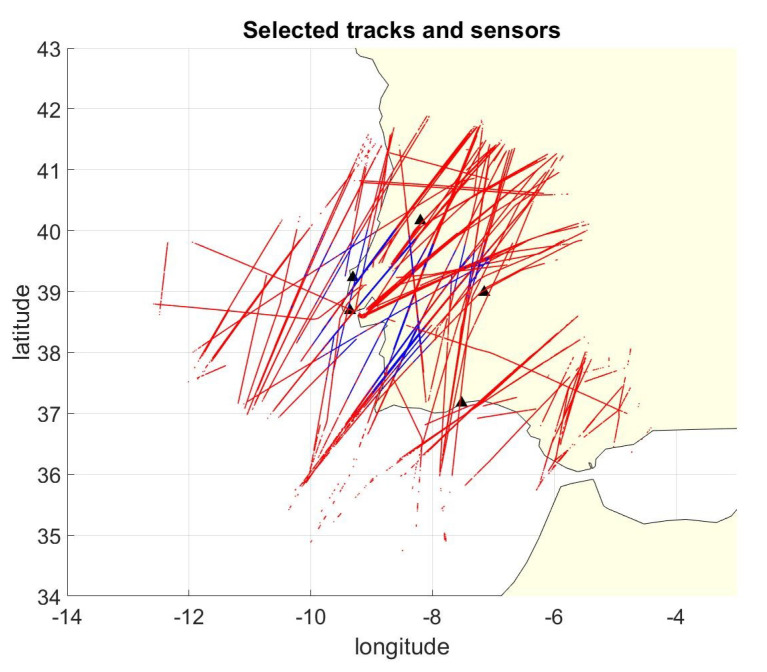
ADS-B tracked routes and selected stations. In the red position, it is not possible to apply the classic MLAT localization algorithm (M<4).

The OpenSky Network is one of the biggest crowd sourced ADS-B networks able to measure and store TOA of each received message, and, in this work, we use the Localization Reference Data Set [45] to evaluate the proposed algorithm performances. A sub-set of the data was selected from the entire data-set and, in particular, only the data coming from five GPS synchronized stations in Portugal/Spain near Lisbon are selected. The recording is one hour long and contains 264,799 ADS-B position messages coming from 163 different aircraft. Figure 2 shows the tracks used for the simulation and the station positions. Note that, in the figure, blue represents positions where more than three stations are in view, red represents positions where less than four stations are in view: for most of the coverage area, the MLAT approach cannot be used to localize the aircraft or to check their ADS-B data with independent localization.

**Figure 3:**
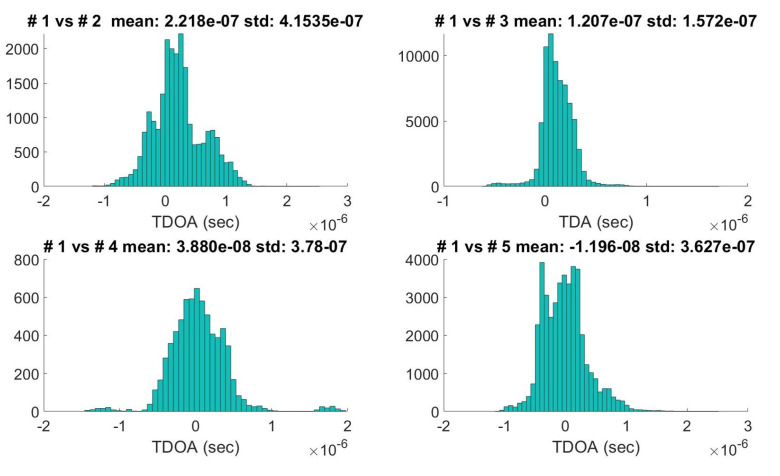
TDOAs means and standard deviations computed for the five stations.

Due to different types of sensors that use different algorithms for TOA computation (for example, some types of receivers refer to the message starting time and others to the message ending time), there is an instrumental delay that must be corrected to compute the TDOA measurements. The whole data-set is used to estimate the covariance matrix $Q_{TDOA}$ and the fixed instrumental delay of each combination of stations (computing the standard deviation, and the mean of all the $TDOA_{i,j}$ pairs). The $Q_{TDOA}$ was computed with Equation (16) setting $\sigma_{1}=\ldots =\sigma_{M}=c\cdot 0.7\cdot 5\cdot 10^{-7}$ s (the coefficient 0.7 is used to refer to TOA instead of TDOA, assuming the measurements error for the two stations have the same distribution). Examples of calculated TDOA distributions (Station #1 versus the others) are reported in Figure 3. Finally, the ADS-B position error covariance matrix was fixed setting $\sigma_{x}=\sigma_{y}=\sigma_{z}=40$ m.

The following sections report the evaluations for the proposed algorithm in case of:

- GNSS/ADS-B/tampering attack that introduces a bias in the ADS-B position;
- ADS-B spoofing that injects fake aircraft following fake but realistic trajectory;
- GNSS jamming introducing additional errors in the ADS-B position information.

In any case, the OpenSky database was intended as the nominal case, and the attacks were simulated applying the models described before.

### 4.1. Gnss/ADS-B/Tampering Attacks

In this section, the algorithm performances for all the attacks that can be emulated adding a bias on the ADS-B position are evaluated. The attack was simulated introducing a bias having a given magnitude and random direction at a given time for each aircraft in the database. For each airplane, after the track initialization phase of the EKF, for every incoming message, a bias with a random direction was generated, and the tests were performed. Then, the track was updated considering the ADS-B positional data without the bias (in this way, the next step can still be used to perform a new test). This simulation tested the ability of the proposed algorithm to detect a step of the ADS-B/TDOA incoming data with respect to the aircraft track generated by the EKF.Different values for the bias magnitude (from 400 m to 1000 m) and th1/th2 (from 0.5 to 4 times the threshold needed to theoretically fix $P_{fa}$ to 0.001 computed with Equation (25)) are tested, and, for each combination of the parameters, the probability of detection and the probability of false alarm were computed.

**Figure 4:**
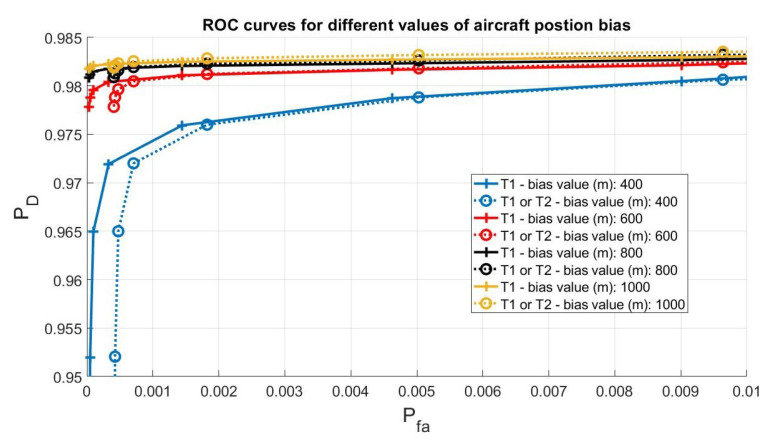
Receiver Operating Characteristic for T1 and T2 different bias values.

Receiver Operating Characteristic (ROC) for different bias values are reported in Figure 4. It is possible to note that the algorithm is able to detect the attack with a high probability of detection and low probability of false alarm also in case of a small bias (400 m) in the ADS-B position; for example, the T1 test (that first test on the innovation) reaches a $P_{fa}=5\times 10^{-5}$ with $P_{D}>0.95$.

Note also that the second test (the one that use the TDOA) does not introduce performance improvements and, on the contrary, using both the tests (T1 or T2 curves) slightly reduces the $P_{fa}$. The T2 test performs worse than the T1 test in this class of attack because, recalling its definition from Equation (32) and Table 1, it depends on the TDOA measurement noise covariance matrix and network geometry. In the specific case of a crowd-sourced network, the TDOA measurements usually are very noisy, and the test is not able to detect a small deviation from the aircraft trajectory. It follows that, for this specific set of data, the T2 test becomes unnecessary for this attack, but mandatory for false track injection, as will be shown later. This condition can change in the case of a network of professional receivers with optimized geometry.

### 4.2. ADS-B Spoofing Attack, Fake Track Injection

In this section, the capability of the algorithms to detect an injection of totally fake tracks is evaluated. The simulation still exploits the previous database but now the ADS-B position of the incoming signal was considered transmitted from an ADS-B spoofer in a given fixed position on the ground. New TDOA measurements consistent with the spoofer position were generated, and the algorithm was applied to track the aircraft. As before, at each step, the ADS-B test (T1) and the TDOA test (T2) were done. In this condition, the test T1, as expected, was not able to detect the attack reaching a maximum $P_{D}$ of 0.1. This happens because the ADS-B position follows a realistic trajectory, and the track/ADS-B position test is not able to detect the attack. On the contrary, in this condition, the TDOA test (T2) has a $P_{D}>0.98$ for any tested threshold value, reaching a probability of false alarm $3\times 10^{-4}$.

Considering the complete algorithm (T1 or T2 to rise an alarm), the probability of detection does not change and the probability of false alarm grows to $4\times 10^{-4}$. Concluding, in any case, if an ADS-B spoofer starts transmitting message with fake positions, the complete algorithm has a high probability to detect it.

### 4.3. GNSS Jamming Attack

GNSS jamming attack was simulated introducing a jammer in a random position and at a random time instant. Once the jammer was introduced, the Probability of Detection and the Probability of False Alarm were computed.

This time, the attack was considered detected if an alarm was raised within 60 s from the time of introduction of the jammer; otherwise, the attack was considered not detected: all the ADS-B messages received within 60 s were used for the test. The jammer was simulated using Equation (9), fixing *a* and *b* so that the additive contribution of to the noise ($\sigma_{Jamming}$) was 200 m for a target closer than 10 km, decreasing linearly until a range of 100 km where it becomes 0 m.

Three different cases for raising an alarm were considered: at least one detection in 60 s, at least two detection in 60 s, and at least three detections in 60 s. This was done to reduce the false alarm rate.

**Figure 5:**
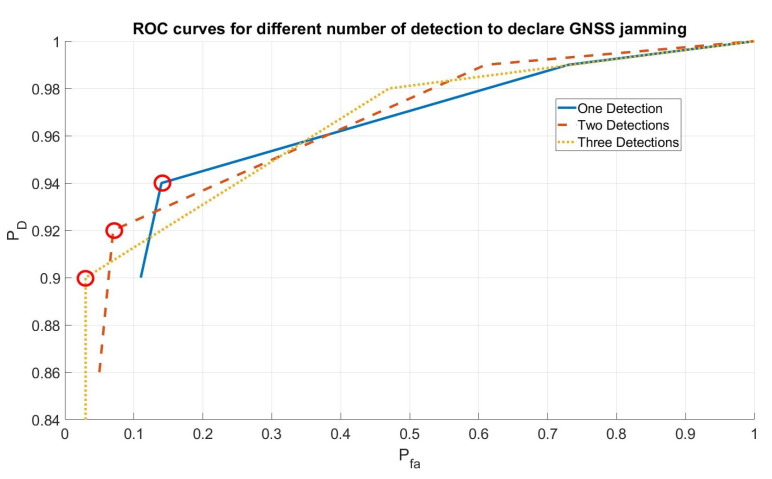
Receiver Operating Characteristic for different numbers of detections.

**Figure 6:**
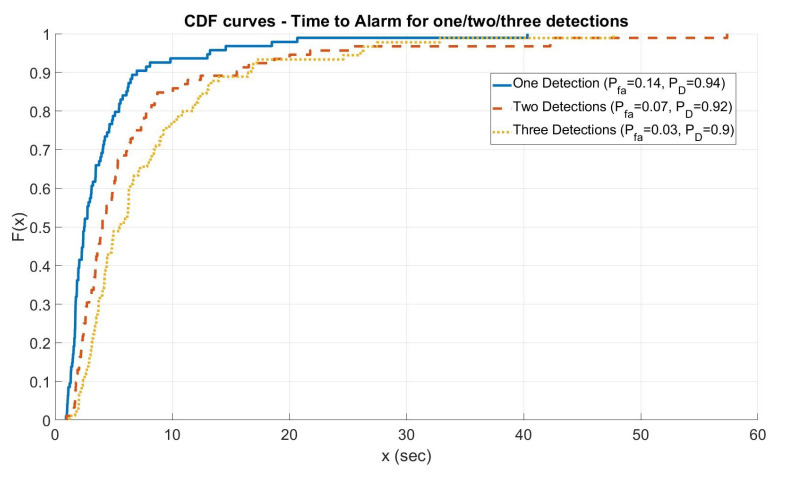
Cumulative Distribution Function for the Time to Alarm in the three different cases.

Figure 5 shows the ROC curves computed for the three different cases varying the threshold values of algorithms th1 and th2. In any case, the alarm was always raised from the first step of the algorithm T1. As shown in the figure, the algorithm is able to assure a good compromise between $P_{D}$ and $P_{fa}$. Furthermore, for this type of attack, the Time to Alarm was also evaluated. Figure 6 shows the cumulative distribution function for the time to alarm in the three different cases (one/two/three detections) for the point highlighted with a circle in Figure 5 (the knees of the curves that are, approximately, the best compromise between $P_{d}$ and $P_{fa}$): the algorithm assures a time to alarm lower than 15 s in 80% of the cases.

## 5. Conclusions

In this work, different models for different types of attacks on the ADS-B and GNSS systems are developed. These models are used to evaluate a two-step EKF algorithm in an ADS-B sensor network to detect cyber-attacks on. The algorithm shows good performances for all the tested conditions and provides alarms for all the cases: the on-board tampering, the ADS-B message injection, GNSS spoofing, and GNSS jamming. This capability was tested with real data coming from the OpenSky Network and simulating the different attacks; we obtained a very high probability of detection and a low probability of false alarm for almost all the cases. In more detail, the proposed algorithm is able to detect, in real time, a small step in the target trajectory also in the case of biases of only 400 m, with a probability of detection higher than 0.97 and a probability of false alarm lower than $3\times 10^{-4}$. The algorithm is also able to detect totally false tracks, not confirmed from TDOA measurements, with a probability of detection higher than 0.98 and the same probability of false alarm as before. Finally, in the case of GNSS jamming, the algorithm shows a probability of detection of the jamming attack of about 0.9 with a probability of false alarm equal to 0.03, detecting the jammer in less than 15 s in 80% of the cases.

Moreover, due to the diffuse and pervasive presence of the ADS-B stations used for the crowd sourced network, the proposed method can be used to monitor both the ADS-B and GNSS systems to detect false ADS-B traffic injection, GNSS spoofing, and jamming without the need for new hardware installation. All the ADS-B equipped aircraft together with the ADS-B ground stations can be considered as a global warning network to detect the cyber-attacks, in particular GNSS attacks.

Last, but non least, alarms generated in this contest can be also used to warn all the other users that use the GNSS for other applications.

## Figures and Tables

**Table 1:** Proposed Algorithm.

**Input:** $s(t_{n})$: aircraft state vector at time $t_{n}$; $S(t_{n})$: state covariance matrix at time $t_{n}$; G: transition matrix; W: process noise covariance matrix; f(·): TDOA formulation, quadratic equations as derived in Equation (15) $F_{ADS-B}$: ADS-B observation matrix as defined in Equation (29); $F_{TDOA}$: TDOA observation matrix as defined in Equation (30); $x_{ADS-B}\left(t_{n}\right)$: ADS-B observation vector at time $t_{n}$; $\mathrm{TDOA}(t_{n})$: TDOA observation vector at time $t_{n}$; $Q_{ADS-B}$: covariance matrix of the ADS-B position error; $Q_{ADS-B}$: covariance matrix of the TDOA measurements noise; the hat operator ^, means prediction for time $t_{n+1}$ using information of time $t_{n}$. **Output:** $s(t_{n+1})$: aircraft state vector at time $t_{n+1}$; $S(t_{n+1})$: state covariance matrix at time $t_{n+1}$; alarms: T1, T2;
**Prediction** predict the state for the time instant of new measurements: $\hat{s}\left(t_{n+1}\right)=Gs\left(t_{n}\right)$; predicted the state covariance matrix: $\hat{S}\left(t_{n+1}\right)=GS\left(t_{n}\right)G^{t}+W$; **ADS-B Innovation test** compute the innovation: $y_{ADS-B}\left(t_{n+1}\right)=x_{ADS-B}\left(t_{n+1}\right)-F_{ADS-B}\hat{s}\left(t_{n+1}\right)$; compute the innovation covariance matirx: $R_{ADS-B}\left(t_{n+1}\right)=F_{ADS-B}\hat{S}\left(t_{n+1}\right){\left(F_{ADS-B}\right)}^{t}+Q_{ADS-B}$; compute the test: $w_{ADS-B}={\left(y_{ADS-B}\left(t_{n+1}\right)\right)}^{t}R_{ADS-B}\left(t_{n+1}\right)y_{ADS-B}\left(t_{n+1}\right)$; if $w_{ADS-B}>th_{1}$ then rise alarm T1; **Updating with ADS-B data** compute Kalman gain: $K_{ADS-B}\left(t_{n+1}\right)=\hat{S}\left(t_{n+1}\right){\left(F_{ADS-B}\right)}^{t}{\left(R_{ADS-B}\left(t_{n+1}\right)\right)}^{-1}$; update the aircraft state vector: $s\left(t_{n+1}\right)=\hat{s}\left(t_{n+1}\right)+K_{ADS-B}\left(t_{n+1}\right)y_{ADS-B}\left(t_{n+1}\right)$; update the state covariance matrix: $S\left(t_{n+1}\right)=\left(I-K_{ADS-B}\left(t_{n+1}\right)F_{ADS-B}\right)\hat{S}\left(t_{n+1}\right)$; **TDOA Innovation test** compute the new innovation: $y_{TDOA}\left(t_{n+1}\right){)}^{t}=\mathrm{TDOA}\left(t_{n+1}\right)-f\left(\hat{s}\left(t_{n+1}\right)\right)$; compute the innovation covariance Matrix: $R_{TDOA}\left(t_{n+1}\right)=F_{TDOA}S\left(t_{n+1}\right){\left(F_{TDOA}\left(t_{n+1}\right)\right)}^{t}+Q_{TDOA}$; compute the test: $w_{TDOA}={\left(y_{TDOA}\left(t_{n+1}\right)\right)}^{t}R_{TDOA}\left(t_{n+1}\right)y_{TDOA}\left(t_{n+1}\right)$; if $w_{TDOA}>th_{2}$ then rise alarm T2; **Updating with TDOA measurements** if $w_{TDOA}<th_{3}$ then compute the new Kalman gain: $K_{TDOA}\left(t_{n+1}\right)=S\left(t_{n+1}\right){\left(F_{TDOA}\left(t_{n+1}\right)\right)}^{t}{\left(R_{TDOA}\left(t_{n+1}\right)\right)}^{-1}$; update the aircraft state vector: $s\left(t_{n+1}\right)=s\left(t_{n+1}\right)+K_{TDOA}\left(t_{n+1}\right)y_{TDOA}\left(t_{n+1}\right)$; update the state covariance matrix: $S\left(t_{n+1}\right)=\left(I-K_{TDOA}\left(t_{n+1}\right)F_{TDOA}\left(t_{n+1}\right)\right)S\left(t_{n+1}\right)$;

## Data Availability

Not applicable.
